# An event-based architecture for solving constraint satisfaction problems

**DOI:** 10.1038/ncomms9941

**Published:** 2015-12-08

**Authors:** Hesham Mostafa, Lorenz K. Müller, Giacomo Indiveri

**Affiliations:** 1 Institute for Neuroinformatics, University of Zurich and ETH Zurich, Winterthurerstrasse 190, CH-8057 Zurich, Switzerland

## Abstract

Constraint satisfaction problems are ubiquitous in many domains. They are typically solved using conventional digital computing architectures that do not reflect the distributed nature of many of these problems, and are thus ill-suited for solving them. Here we present a parallel analogue/digital hardware architecture specifically designed to solve such problems. We cast constraint satisfaction problems as networks of stereotyped nodes that communicate using digital pulses, or events. Each node contains an oscillator implemented using analogue circuits. The non-repeating phase relations among the oscillators drive the exploration of the solution space. We show that this hardware architecture can yield state-of-the-art performance on random SAT problems under reasonable assumptions on the implementation. We present measurements from a prototype electronic chip to demonstrate that a physical implementation of the proposed architecture is robust to practical non-idealities and to validate the theory proposed.

## Abstract (web summary)

Constraint satisfaction problems are typically solved using conventional von Neumann computing architectures, which are however ill-suited to solving them. Here, the authors present a prototype for an event-based architecture that yield state of the art performance on random SAT problems.

## Graphical abstract

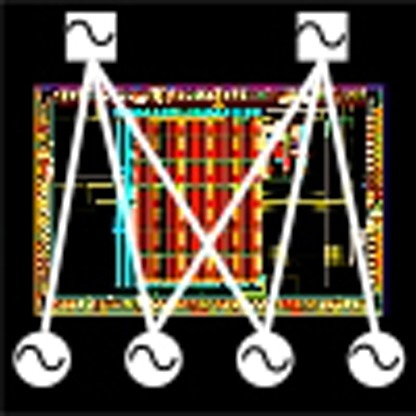

Constraint satisfaction problems (CSPs) are a fundamental class of problems in computer science with wide applicability in areas such as channel coding^1^, circuit optimization^2^ and scheduling^3^. Algorithms for solving CSPs are typically run on classical von Neumann computing platforms that were not explicitly designed for these types of problems. This paper addresses the question: how can we implement a more efficient computing substrate whose architecture and dynamics better reflect the distributed nature of CSPs?

Many dynamical systems that have been proposed for solving CSPs violate the ‘physical implementability' condition^4^,^5^,^6^. Non-physicality arises from the use of variables that can grow without bounds as the system is searching for solutions. On the other hand, there is a long, well-established tradition of studying physically realizable dynamical systems, for example, in the form of artificial neural networks, to solve CSPs or ‘best-match problems'^7^,^8^. Early attempts in this field used attractor networks, such as Hopfield networks^9^, to solve NP hard (non-deterministic polynomial-time hard) problems such as the travelling salesman problem^10^,^11^. These attractor networks, however, would often get stuck at locally optimal solutions. To overcome this problem, stochastic mechanisms were proposed^12^,^13^, which require explicit sources of noise to force the network to continuously explore the solution space. While noise is an inextricable part of any physical system, dynamically controlling its power to balance ‘exploratory' versus ‘greedy' search, or to move the network from an exploratory phase to a greedy one according to an annealing schedule, is not a trivial operation and puts an additional overhead on the physical implementation.

Here we present a mixed analogue/digital hardware architecture whose dynamics execute an efficient search for CSP solutions without the need for external sources of noise. For this reason, the architecture can be easily and efficiently implemented using complementary metal-oxide semiconductor very-large-scale integration (VLSI) electronic circuits. In the proposed architecture, each variable in a CSP is represented by a node consisting of an analogue oscillator and a state-holding asynchronous digital circuit. To achieve robust and scalable computation, the nodes communicate using digital pulses, or events. This combination of analogue and digital circuits running in a hybrid continuous/event-driven mode avoids many of the problems that affect pure analogue VLSI systems such as susceptibility to noise, degradation of analogue signals during storage and communication, and signal restoration/refresh issues. Since the oscillators are realized using analogue circuit, when fabricated, they exhibit incommensurable natural frequencies, that is, frequencies that are not rational multiples of each other. Our architecture relies on the non-repeating phase relations among these incommensurable analogue oscillators to drive the search for optimal solutions, rather than making use of external sources of noise or relying on random fluctuations. We show that the architecture naturally reproduces the dynamics of stochastic local search (SLS) algorithms. These algorithms are typically incomplete, that is, they cannot prove that a solution does not exist for unsatisfiable problems. Our main contributions are a physical architecture with novel dynamics for solving CSPs, together with massively parallel reformulations of well-established CSP algorithms so that they can be efficiently instantiated on the proposed architecture. We present results from an implementation of this architecture on a prototype VLSI chip. Our results expose a surprising relation between the dynamics of coupled multi-stable oscillators and the search for CSP solutions and highlight a novel mode of distributed, parallel, mixed analogue/digital computation that can form the basis of various hardware/physical systems for solving CSPs.

## Results

### Description of the architecture

**Figure 1:**
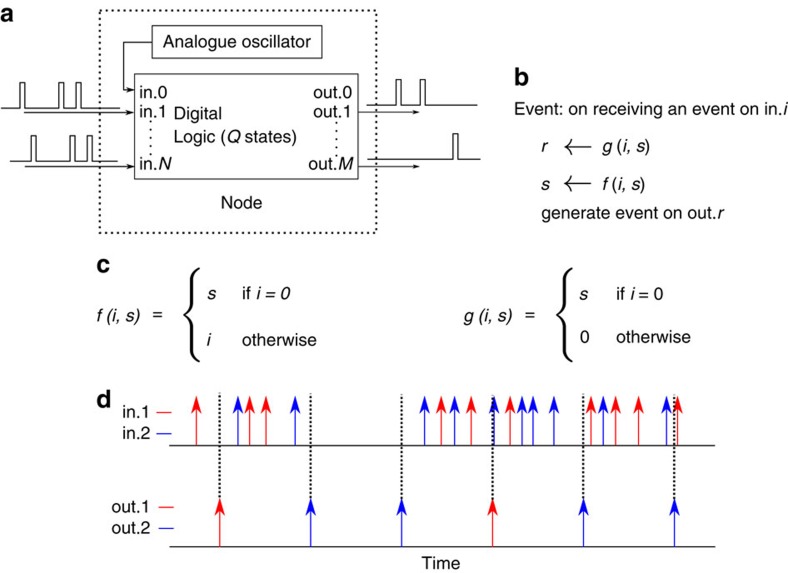
Building blocks of the proposed architecture (**a**) General form of the computational unit in our architecture. This computational unit, or node, is composed of asynchronous state-holding digital logic, and an analogue oscillator that generates a stream of events. The digital logic is event driven and changes its internal state in response to events on its input ports ‘in.0' to ‘in.*N*'. The node can generate an event on one of the output ports in response to input events. (**b**) Formal description of a node. On an input event on port ‘in.*i*', the digital logic evaluates the index of the output port *r*, updates its state *s* and generates an event on output port ‘out.*r*' in that order according to the functions *f* and *g*. Events from the internal oscillator always arrive on the ‘in.0' port. An output event on the ‘out.0' port is discarded, that is, the node does not generate an output event if *r*=0. (**c**) Definition of the *f* and *g* functions for an example binary node with two internal states, two input ports and two output ports (*N*=*M*=*Q*=2). (**d**) Simulation of the example node showing its input and output event streams. The node generate an output event for each event from the periodic internal oscillator. The output events reflect the identity of the last input event the node received.

The proposed event-based architecture for solving CSPs employs a network of nodes that communicate via digital events. A node is shown schematically in Fig. 1a. Each node has *N* externally accessible input ports, one internal input port, *M* output ports and one dummy output port. The analogue oscillator in the node generates a continuous stream of digital events that are sent to the node's internal port: ‘in.0'. The digital logic in the node has an internal state *s*, which can take one of *Q* possible values. On the arrival of an event on any of the input ports, the node's digital logic evaluates the index of the output port to which it should send the event based on the index of the triggered input port and on the current state of the digital logic; it updates its internal state; and it transmits the event via the output port selected (Fig. 1b). Selection of the ‘dummy' output port ‘out.0' is equivalent to suppressing the event. The digital logic is fully described by the event routing function *g* and the state update function *f*, which are both deterministic. Given their analogue nature, the natural frequencies of the oscillators in the different nodes are not rational multiples of each other. Due to fabrication-induced mismatch, different oscillators realized on a VLSI chip will have incommensurable natural frequencies. It is important that coupling between these physical oscillators be kept at a minimum so as to minimize the chance of phase locking.

For solving CSPs, a subset of the nodes in the network will represent the actual problem variables, while others will represent helper variables that encode other problem-relevant quantities (for example, whether a constraint is satisfied or not). The value of a variable/node at a point in time is the index of the output port on which the node emitted its last event. Thus, a variable/node with *M* output ports can have *M* possible values. The output port of one node can connect to the input ports of one or more nodes and one input port can receive events from multiple output ports. One output port cannot be connected to multiple input ports on the same node. In the following sections, we describe how to connect nodes/variables together and how to define the nodes/variables behaviour (the *f* and *g* functions) so as to solve a number of hard CSPs. The procedure to map a CSP to this distributed architecture depends on the type of the CSP but in general, the mapping is done so that the distributed and parallel dynamics of the network of nodes tries to put the problem variables/nodes in a state where their outputs satisfy all the constraints.

Figures 1c and 1d show the definition and illustrate the behaviour of an example node that has two input ports, two output ports and two possible internal states (*N*=*M*=*Q*=2). The state of the example node/variable is the index of the last external event it received and the node/variable advertises its state by generating an event on one of the output ports when it receives an event on the internal port ‘in.0' as shown in Fig. 1c (we refer to this as ‘updating'). Assume this example node is the target node receiving events from multiple sources nodes. Since it is only the last received event that determines the value advertised by an updating node, the phase relations between the analogue oscillators in the network determine which of the source nodes generates the decisive event that determines the event generated by the target node. This would be the source node that updated just before the target node updates. The phase relations are continuously changing in an aperiodic manner since the oscillation frequencies are incommensurable. The shifting phase relations thus continuously change which source node manages to influence the output events of the target node.

For the node described in Fig. 1c, assume *N*_1_ nodes with frequencies 
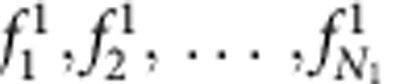
 are sending events to its ‘in.1' port and *N*_2_ nodes with frequencies 
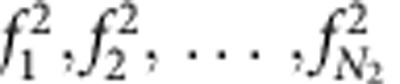
 are sending events to its ‘in.2' port, the fraction of 1 events generated by the target node converges to 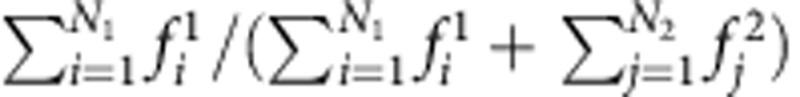 if observed for a long enough time. Assuming the differences in oscillator frequencies of the source nodes are small, the latter expression can be written as 
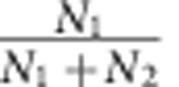
. Thus, the more nodes that try to force a target node to a particular value, the more likely the target node is to output that value, yet there is always a chance that even a single source node that is in conflict with the majority will update just before the target node updates, thereby causing the target node to go against the majority influence. As we will show in the next sections, this behaviour can be exploited to allow the network to escape from local minima where flipping a single variable/node may increase the number of violated constraints. However, a node will never take a value that is in conflict with all incoming influences which is why the globally optimal state is stable. We show that this mostly greedy, but sometimes exploratory, behaviour can be exploited to efficiently solve a variety of hard CSPs.

In contrast to simulated annealing, there is no temperature parameter that balances the greedy versus the exploratory aspect of the network behaviour. The exploration of the solution space is not noise driven but, rather, it is driven by the continuously changing, non-repeating order of event generation. This has the advantage that no cooling schedule is needed and there is no artificial separation between an exploratory (high temperature) phase and a greedy (low temperature) phase. This enables the problem to be dynamically changed and new constraints added without having to restart any cooling schedule. Due to the incommensurable oscillation frequencies, the network trajectory is aperiodic, so the system does not get stuck in loops. One disadvantage, however, of using deterministic incommensurable oscillators to drive the search is that we cannot derive probabilistic convergence results as in simulated annealing.

### Boolean satisfiability problems

Let **X**={*x*_1_,…,*x*_*N*_} be a set of Boolean variables. A literal is either a variable or its negation. The solution to a Boolean satisfiability or K-SAT problem is the variable assignment that satisfies the logical expression involving the variables of **X**:


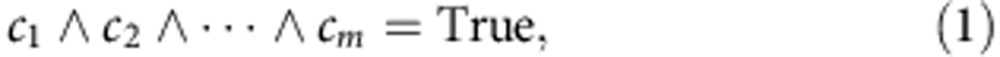


where the clause *c*_*i*_ is the disjunction of *K* literals. K-SAT for *K*≥3 is NP complete^14^.

A highly efficient algorithm for solving random SAT problems is the probSAT algorithm^15^, winner of the parallel random track of the 2014 satcompetition^16^. probSAT iteratively modifies a variable assignment by choosing a random unfulfilled clause *c*_*u*_ and changing the assignment of (‘flipping') a random variable *x*_*f*_ in *c*_*u*_, thereby fulfilling *c*_*u*_. The choice *x*_*f*_ is governed by a heuristic function *f*(*m*,*b*), where *m* (the ‘make' heuristic) is the number of clauses that are newly fulfilled when *x*_*f*_ is flipped and *b* (the ‘break' heuristic) is the number of clauses that are newly unfulfilled when *x*_*f*_ is flipped. The heuristic function is renormalized into a probability over the available choices and *x*_*f*_ is chosen according to these probabilities. The heuristic function *f*(*m*,*b*) can take several different forms. In our benchmarks, we use the particularly effective ‘exponential' form:


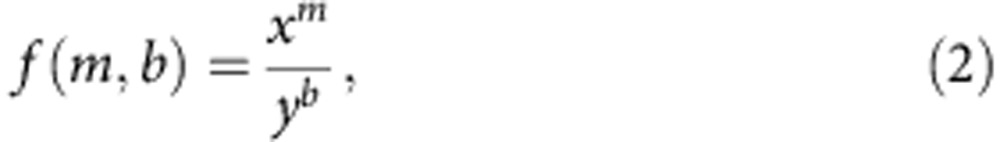


where *x* and *y* are parameters. If no solution is found after *n*_max_ flips, all variables are set to new random values, that is, the algorithm is restarted.

We consider K-SAT for *K*∈{3,4,5}. We map the probSAT algorithm with only the ‘break' heuristic to our architecture using two types of nodes: nodes representing variables and nodes representing constraints/clauses. Each variable node has two states. It updates and advertises its state (by generating an event on one of its two output ports) whenever it receives an event from a clause node. In addition, it advertises its state whenever it receives an event from the internal oscillator.

**Figure 2:**
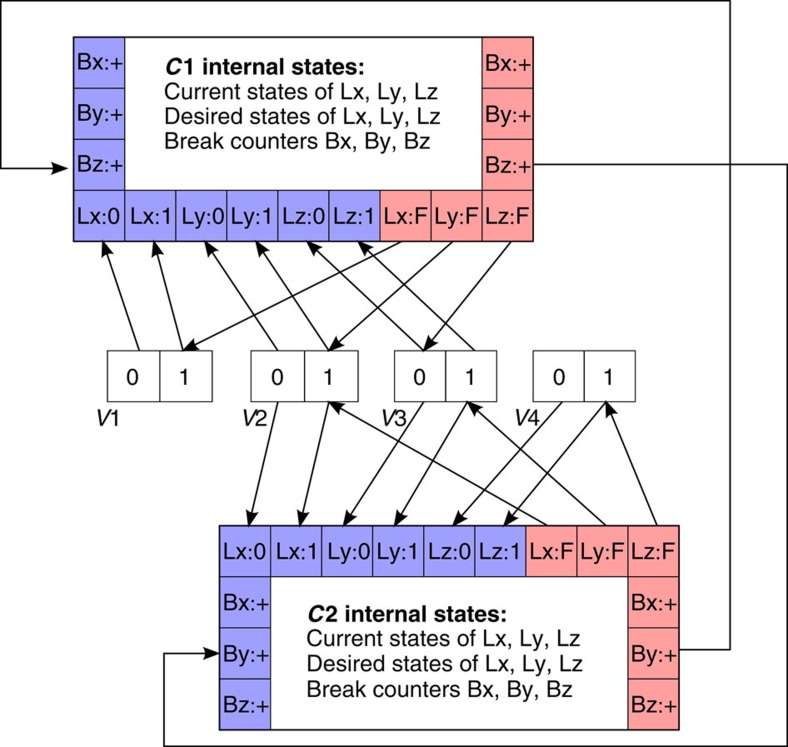
Sample network implementing probSAT. Network corresponding to the example SAT problem *C*1∧*C*2 where *C*1=(*V*1ν*V*2ν−*V*3) and *C*2=(*V*2ν*V*3ν*V*4). For the constraints *C*1 and *C*2, the squares at the edge of the box indicate input ports (blue) and output ports (red). Events are routed along the arrows. Each unfulfilled constraint node periodically choose a variable in its domain to flip. The chosen variable is the one with the lowest break count, that is, the variable that will cause the smallest number of other clauses/constraints to be unfulfilled when flipped. A constraint node updates its break counters based on the events it receives from other constraint nodes that have one or more variables with opposite polarity in common.

Figure 2 shows a sample 3-SAT network. When the clause node receives a break event (event arriving on one of its break input ports, one corresponding to each variable), it increments the corresponding break counter. On events from the internal oscillator, a clause node evaluates what state the connected variables have last advertised. If there is no variable in a fulfilling state, the clause node sends an event to flip the variable with the smallest associated ‘break' count and sends a ‘break' event to every clause node in which this variable appears with the opposite polarity to indicate that the flipped variable is the only variable keeping the constraint fulfilled. If there is exactly one fulfilling variable, the clause node only sends a ‘break' event for that variable; every *r*th ‘break' event is skipped and not sent where *r* is a fixed parameter. If there is more than one fulfilling variable, the clause node does not send out any events. The break counters are reset after each event from the internal oscillator.

An unfulfilled clause node thus always chooses to flip the variable with minimal break count (with ties resolved according to a fixed variable ordering). The skipping of some break events implements a ‘softening' of this hard minimum function. This flip heuristic is deterministic and simpler than the heuristic employed by standard probSAT.

We also implement a functional replacement of the random reinitialization: if after *n*_max_ variable updates, no solution has been found, all literals receive a ‘flip' message. This is not a random reinitialization, but it is a method of forcing the algorithm into a distant part of the solution space.

We compare the performance of the network to that of the standard (sequential) probSAT algorithm^15^. Our aim here is to show that the modifications we introduced to tailor probSAT to our architecture have not degraded its performance. We evaluate the network performance in several different cases: The ‘ideal' case where events are transmitted instantly and never lost; and a ‘non-ideal' case where events have a delay uniformly distributed between 0 and 10% of the node oscillation period and a 10% chance to get lost completely. The non-ideal case simulates the imperfections of an actual physical implementation where event delivery is neither instantaneous nor guaranteed. We also consider the case in which nodes update at a pseudo-random order, which is equivalent to having the oscillator in each node generate a Poisson instead of a periodic event train. This pseudo-random update ordering more closely matches the pseudo-random selection of unsatisfied clauses in the standard probSAT algorithm.

As benchmarks, we use uniform-randomly generated 3-, 4- and 5-SAT instances of various sizes and (in the case of 4- and 5-SAT) with various clause densities *α* (the ratio of clauses to variables). Different values of *α* have been shown to correspond to different geometrical organizations of the solution space^17^; we have chosen the values of *α* so that they lie in the different hard regimes.

We cannot give a comparison with state-of-the-art benchmarks because our software simulator is only able to simulate 10 cycles for each node per second if used to simulate networks implementing large modern benchmarks. On the basis of the solution times of standard probSAT, 10^6^–10^9^ cycles are needed to solve a single modern benchmark problem (that is, 1–1,000 days to simulate the corresponding network). As an alternative, we evaluate the performance on various problem sizes to ensure that the network performance scales equally well as standard probSAT.

**Figure 3:**
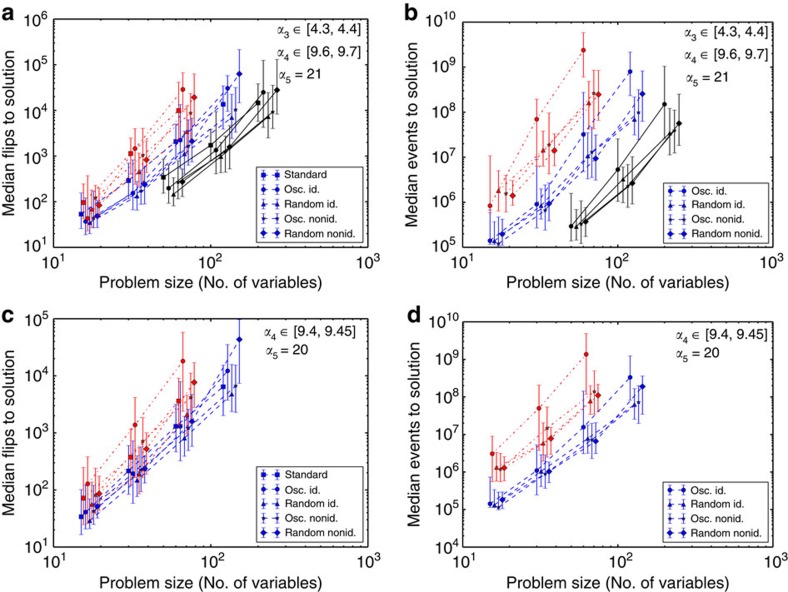
Performance of the network implementation of probSAT. (**a**)–(**d**) Median flips and median events to solution on 3-, 4-, and 5-SAT problems for different solution strategies: for standard probSAT (standard), networks with instantaneous and guaranteed event delivery (id.), networks with event loss and delays (nonid.), networks where node event generation is periodic (osc.) and networks where node event generation is Poissonian (random). Error bars show first and third quartiles (that is, half of the data lies within the error bars). Black solid lines show 3-SAT (*n*_var_∈[50,100,200]), blue-dashed lines show 4-SAT (*n*_var_∈[15,30,60,120]) and red-dash-dotted lines show 5-SAT (*n*_var_∈[15,30,60]). To avoid overlap, some data points are slightly shifted along the *x* axis. The clause densities, *α*_3_,*α*_4_,*α*_5_ used for 3-, 4-, and 5-SAT, respectively, are shown on the plots. For 4- and 5-SAT, two different caluse density values were tested, corresponding to different geometrical arrangements of the solution spaces (no multiple regimes exist for 3-SAT)^17^. Each data point was obtained by solving 100 instances. Note the logarithmic scales on both axes. Standard probSAT and our network implementation scale similarly well with the problem size.

Figure 3 shows the median number of variable flips required to reach the solution; this measure shows the effectiveness of a SLS algorithm in an implementation independent way. The various implementations of probSAT show very similar scaling behaviour in all cases; it is therefore reasonable to assume that for large problems the network performs as well as the standard algorithm in terms of number of flips to solution. To match the 1–10 Mega-Flips per second that a standard current CPU achieves when running probSAT, each node in our network version would need an analogue oscillator of frequency around 2 MHz (in the network, approximately five flips happen each cycle due to parallelism).

A potential bottleneck in our proposed hardware architecture is the event routing fabric. We can calculate the approximate routing rate necessary to match the standard implementation of probSAT by dividing the number of events to solution (Fig. 3) by the median runtime of standard probSAT, which was 10 ms for the hardest 4-SAT problems. This yields a required combined event routing rate of around 100 billion events per second. Note that a single event generated by a node is typically dispatched to multiple nodes. An event generated by a node is thus typically split into multiple events, each targeting a single node. Each of these ‘split' events is separately counted when calculating the required event rate. A 100 billion events per second rate can be achieved if the highly local nature of node communication (which reflects the local nature of the constraint graph) is exploited by a parallel event routing scheme, and if the event routing scheme supports multi-casting that allows a single source event to be efficiently routed to multiple destinations.

Using this approach, we can make similar statements with respect to other SAT solvers: at what oscillation rate and event routing rate need our architecture be implemented to match the solution times of other solvers on sample problems (in this case, however, the comparison does not say anything about solution times on bigger problems, because there is no structural similarity between the solvers). For two other well-known solvers, minisat^18^ and survey propagation^19^, we study the solution times of the hardest 4-SAT problems (for smaller problems the overhead starting the solvers would skew results unfairly in our favour); in the case of survey propagation, we only took into account the times to correctly converged solutions. Unsurprisingly, probSAT is somewhat faster; to match minisat on these problems, we require an average oscillator frequency of 0.2 kHz and an event routing rate of 0.1 billion events per second, and to match survey propagation, an oscillator frequency of 0.4 MHz and a 20 billion events per second event routing rate is needed.

Surprisingly, the non-ideal network performs better than the ideal one. Losing/delaying events might increase network efficiency by making it more exploratory as clauses now have imperfect information about the state of the variable nodes. The comparison between the pseudo-random update ordering and the ordering induced by the incommensurable oscillators shows that a pseudo-random ordering is slightly more efficient.

The architecture we present in this paper is well suited for implementing SLS algorithms such as probSAT as it can exploit the non-repeating phase relations among the incommensurable oscillators as a source of non-repeating noise that drives the search. Local search algorithms are typically incomplete, that is, they cannot prove that a solution does not exist for unsatisfiable formulae. In Supplementary Fig.1 and Supplementary Note1, we describe how a complete SAT algorithm can be mapped to the proposed architecture. We use an algorithm that is custom tailored to the architecture and that combines an SLS-like search inspired by probSAT with a systematic pruning of the SAT solution tree. The network of nodes implementing the complete algorithm is guaranteed to either find a solution or to generate an event indicating that the problem is unsatisfiable. One disadvantage of this complete algorithm is that it requires certain events to be globally routed to all nodes, thus degrading the efficiency of any parallel event routing scheme that seeks to exploit the local nature of internode communication. These global events are a reflection of the typically centralized and sequential nature of complete algorithms.

**Figure 4:**
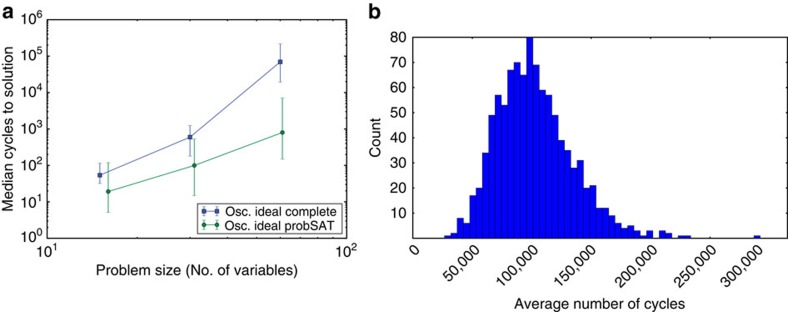
Performance of the network implementing the complete SAT algorithm. (**a**) Median of the average number of oscillation cycles to solution (averaged over the nodes in the network) for the ideal oscillatory network implementing probSAT and for the ideal oscillatory network implementing the complete algorithm when solving random 4-SAT instances with *α*_4_∈[9.4,9.45]. Error bars show first and third quartiles. The network implementing the complete algorithm is slower than the one implementing probSAT when searching for solutions to satisfiable instances. (**b**) Histogram of the average number of cycles (averaged over the nodes in the network) taken by the network implementing the complete algorithm to signal that a 3-SAT instance is unsatisfiable. Network was run once per instance on 1,000 random 3-SAT instances with 100 variables and 430 clauses each.

The network of nodes implementing the complete algorithm is not as efficient as the network implementing the probSAT algorithm when solving satisfiable K-SAT instances. This is illustrated in Fig. 4a where the complete algorithm takes significantly more cycles to find a solution. Although not as efficient as the probSAT network, the network implementing the complete algorithm can detect that a problem is unsatisfiable as shown in Fig. 4b.

### Graph colouring problems

A *k*-colouring for an undirected graph *G* with vertices *V*(*G*) and a set of edges *E*(*G*) is a map *φ*:*V*(*G*)→{1, 2, …, *k*}. In the graph colouring problem, the goal is to find a proper k-colouring *φ*_0_ of *G* where *φ*_0_(*x*)≠*φ*_0_(*y*) for all {*x*,*y*}∈*E*(*G*).

**Figure 5:**
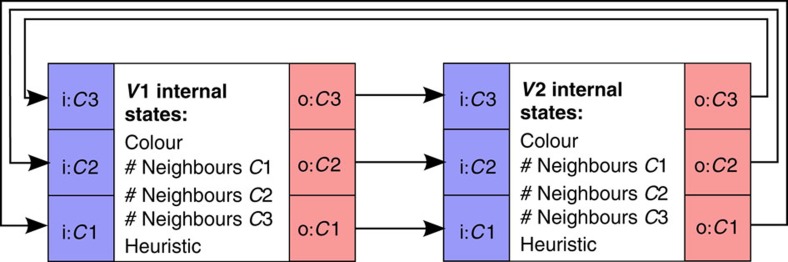
Network solving graph colouring. Network corresponding to the three-colouring of the graph *V*={*V*1,*V*2}, *E*={(*V*1,*V*2)}. The squares at the edge of the box indicate input ports (blue) and output ports (red). Events are routed along the arrows. When two connected nodes represent the same colour, one of them will change its colour on its next internal oscillator event.

To solve a *k*-colouring problem, we map each vertex in the graph to a network node with *k* input ports and *k* output ports as shown in Fig. 5. Whenever the internal oscillator in a node/vertex generates an event, the node advertises its colour by generating an event on one of the *k* output ports. Events from a node/vertex are routed to all its neighbours in the graph. Each node maintains *k* counters that count how many of its neighbours have a particular colour. These counters are incremented when a node receives events from its neighbours. At an internal oscillator event, if the counter corresponding to the current node colour is non-zero (one of the neighbours has the same colour), the node chooses a different colour. If the internal Boolean variable, ‘heuristic', is true, the node chooses the colour with the fewest conflicts (smallest neighbour count). If ‘heuristic' is false, the node chooses the next colour in a fixed arbitrary ordering of colours. The node then resets the *k* counters, flips the ‘heuristic' binary variable and generates an event to advertise its colour. A min conflict heuristic thus alternates with a heuristic-free scheme to update a conflicting node each cycle.

We assessed the performance of this algorithm on several *k*-colouring problems of intermediate difficulty (Table 1) taken from ref. 20 in which a different massively parallel colouring algorithm (gravitational swarm intelligence) was assessed. As in the previous section on Boolean satisfiability, we cannot attempt state-of-the-art-sized problems since the software simulation of a large network takes an infeasibly long time. In terms of average numbers of oscillation cycles to solution, the network compares favourably with gravitational swarm intelligence^20^.

### Prototype VLSI implementation

The prototype VLSI chip that implements a version of the architecture described in this paper is composed of a two-dimensional array of binary nodes that communicate using events. The problem of transmission and routing of asynchronous events has been thoroughly investigated in the neuromorphic engineering literature^21^,^22^. An elegant solution uses the address-event representation (AER) protocol. When a node generates an event on one of its output ports, it executes a handshake protocol with the ‘output AER interface'. The ‘output AER interface' encodes the address of the output port on which the event was generated and transmits the address off-chip using an output bus that has log_2_(*K*_out_) lines. *K*_out_ is the number of possible event sources (the output ports of all the nodes). The array has *K*_in_ possible event targets (the input ports of the nodes), if an event is to be sent to one of these targets, the target address is sent to the ‘input AER interface' on a bus that has log_2_(*K*_in_) lines. The ‘input AER interface' decodes the address and sends an event to the target element by simultaneously activating the correct row and column in the array.

**Figure 6:**
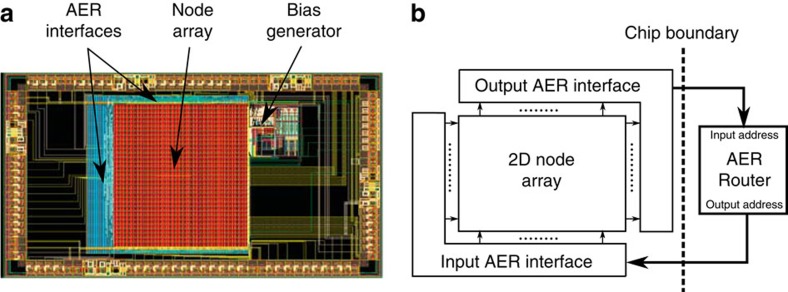
Prototype chip and test system. (**a**) Layout of the minimum size (2*3 mm) prototype chip fabricated using a 180-nm complementary metal-oxide semiconductor process that implements the architecture described in this paper. The 64*32 node array in the middle is surrounded on three sides by the digital asynchronous AER interfaces. An externally programmable bias generation block generates the analogue biases needed by the analogue oscillators. (**b**) The test system. An off-chip event router implemented on a field programmable gate array communicates with the chip AER interfaces to route events from output ports to input ports.

The two-dimensional array on the chip comprises 64*32 binary nodes/variables, that is, nodes/variables with two output ports as shown in Fig. 6a. The chip can be configured so that two, three or four adjacent variables are merged together to realize four-, six- or eight-valued variables, respectively. An *n*-valued variable (*n*∈{2,4,6,8}) has *n* output ports and *n* possible internal states and has 2^*n*^−1 input ports. Physically, a variable has *n* digital input lines on which it receives an *n*-bit binary word encoding the index of the input port receiving the event. The details of the chip nodes are given in the methods section. An off-chip event router implemented on a field programmable gate array communicates with the output and input AER interfaces to route events from nodes/variables output ports to input ports according to a programmable routing table as shown in Fig. 6b.

The analogue oscillator in each node is realized using an analogue integrate and fire neuron^23^ receiving constant current injection. In the methods section, we describe the frequency distribution of the on-chip nodes and analyse internode phase coherence. The node oscillators have different frequencies but there is minor coupling between them which, however, does not amount to phase locking. The exploration of different phase relations, which is crucial to the search scheme employed by the architecture, thus remains intact.

**Figure 7:**
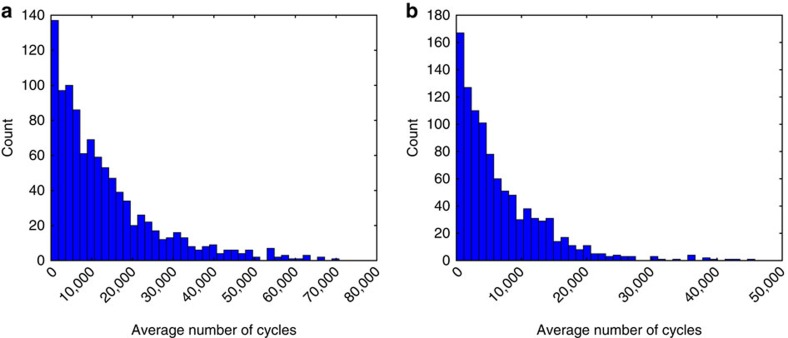
Performance of the prototype chip. (**b**) Histogram of the number of oscillation cycles (averaged over all nodes) needed by the chip to find the solution of a 3-SAT problem with 50 variables and 218 clauses over 1,000 trials. (**b**) Histogram of the number of oscillation cycles (averaged over all nodes) needed by the chip to find the optimal colouring of the 5 × 5 queen graph over 1,000 trials.

Using the node logic on the prototype chip, we implemented a 3-SAT algorithm, which is based on the algorithm from ref. 24. At the end of a cycle, an unfulfilled clause sends an event to flip the last variable in its domain to generate an event. Due to the continuously changing phase relations, an unfulfilled clause effectively chooses almost at random a variable to flip similar to the algorithm in ref. 24. Details of implementing this algorithm on the hardware prototype are given in the methods section. Figure 7a shows a histogram of the average number of oscillation cycles needed to find the solution of an example 3-SAT problem.

The hardware prototype can solve graph colouring problems with up to eight colours. A *k*-colour node/vertex advertises its colour by generating an event on one of its *k* output ports at the end of its internal cycle (when its internal oscillator generates an event). This event is routed to all its neighbouring nodes/vertices in the graph, which causes these nodes to take a colour that is different from the advertised colour. Details of the graph colouring algorithm implemented on the prototype chip are given in the methods section. One difficult graph for this architecture is the ‘5 × 5 queen' graph whose solution is equivalent to finding the non-interfering positions of 5 queens on a 5 × 5 chess board. The average number of cycles needed to find a solution is shown in Fig. 7b.

## Discussion

CSPs have often been examined through the lens of statistical physics^25^,^26^. Within the framework of statistical physics, a CSP is formulated as a distributed system that seeks to minimize the number of frustrated interactions (violated constraints) between its elements. Direct analogies can be established between the ground energy states of physical systems (where frustrated interactions are at a minimum) and solutions to CSPs^27^. The architecture we describe in this paper is fundamentally different from the systems analysed in the framework of statistical physics, yet it captures some of the general features of such systems: the architecture makes use of a large number of locally interacting elements that mutually constrain each other so that the system as a whole tries to go to states where the number of frustrated interactions is at a minimum.

The most distinguishing feature of our system is the mechanism used to explore the solution space. In lieu of random fluctuations, the continuously changing phase relations between incommensurable oscillators are a source of non-repeating fluctuations that can be easily exploited in our event-based architecture to realize efficient search algorithms. Pseudo-random number generators (PRNGs) could have been used to drive the search. Even assuming we could implement an efficient PRNG with a per-node complexity equivalent to an analogue oscillator, PRNGs would require a clock. In each clock cycle, the PRNG scheme could choose one variable/constraint to update at random. This sequential scheme will fail to exploit the distributed and highly local nature of many CSPs (a constraint involves only few variables and a variable is part of only few constraints). This distributed and local nature is precisely what we are trying to exploit with the independent nodes running in parallel and trying to attain consistency in their local neighbourhoods in the constraint graph. The PRNG scheme could update multiple, randomly chosen, variables/constraints per clock cycle. Such batch updates would go against the idea of probSAT, and SLS algorithms in general, which make local moves that change only one variable at a time and propagate its new value before updating the next variable. Since nodes in a distributed architecture need to communicate, by having them communicate in an event-based manner at times governed by simple local analogue oscillators, we obtain (almost for free) non-repeating fluctuations that could be easily exploited to drive a stochastic-like search.

While the architecture is general enough to allow the instantiation of various algorithms for solving CSPs, it is best suited to implementing algorithms of the local search variety in which each variable is iteratively updated based only on local information, that is, based on the state of the constraints in which it is involved.

The digital event-based nature of node communication is key to the architecture's scalability and configurability. These digital pulses can be transmitted and routed using a digital fabric that links together a large number of nodes. In the prototype chip, event routing is done off-chip in a serial manner on a field programmable gate array board^28^. This introduces a serial bottleneck in the otherwise massively parallel operation of the architecture. This serial bottleneck must be eliminated to reap the advantages of the massively parallel operation of the distributed architecture. A distributed event routing architecture that exploits the local nature of event communication (which reflects the local nature of the constraint graphs of many relevant problems) to route events in parallel in multiple local domain is necessary. Configurable and parallel AER routing fabrics have been proposed for use in large-scale neuromorphic systems^29^,^30^ and could be directly adapted for use in an implementation of the described architecture. As shown when solving SAT problems, the architecture is robust to event delays and lost events that relaxes the requirements on the event routing fabric.

In simulation, we showed for the case of random SAT problems that the proposed architecture can run at a surprisingly slow mean oscillation frequency (around 2 MHz) and still attain a time to solution that is comparable to a CPU running at three orders of magnitude higher clock rate. The simple logic operations in the constraint and literal nodes can certainly run at such slow frequencies. These results indicate that the proposed architecture is a more efficient approach to solving SAT problems than conventional CPUs.

Algorithms for solving CSPs are often conceived with the digital von Neumann model of computation in mind. The results presented in this paper highlight an alternative approach that starts with no prior assumptions about the computational model, and seeks to exploit the physical characteristics of the underlying substrate in order to find a solution tailored to the computational problem at hand. In our case, we exploited the natural incommensurability of physical analogue oscillators to derive a distributed novel algorithm for solving CSPs. The resulting physical algorithms naturally admit an efficient implementation on the physical substrate that underlies their derivation. The computing architectures developed using this bottom-up approach, such as the VLSI device we present in this paper, have the potential to achieve considerable performance gains in their target problems compared with conventional purely digital approaches^31^.

## Methods

### Description of the chip node

When an *n*-valued chip node (*n*∈{2,4,6,8}) receives an event on one of its 2^*n*^−1 input ports, say port *i*, The 1s in the binary representation of *i* denote the allowable internal states that the variable can take. The node has *n* possible internal states and an event on one of the 2^*n*^−1 input ports can thus decide which non-empty subset of these states are allowed. If multiple states are allowed, the variable stays at its current state if the current state is one of the allowed states, otherwise it goes to the lowest index allowed state. Let *i*(*p*) be the *p*th bit of *i* where indexing starts at 1, the state update function *f* is thus:


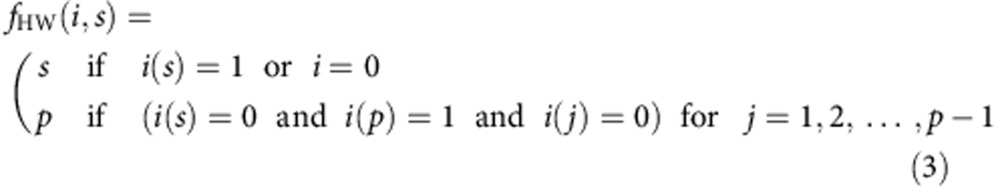


The node/variable generates an event only when it receives an event from the internal oscillator on port 0. The event is generated on the port corresponding to the currents state. The event routing function *g* is:


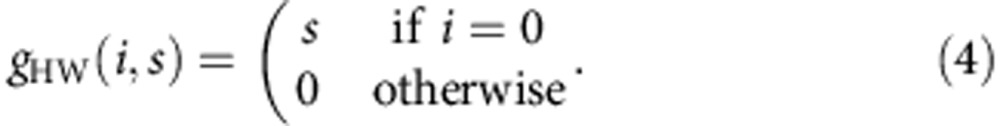


### Frequency distribution of chip nodes and internode coherence

**Figure 8:**
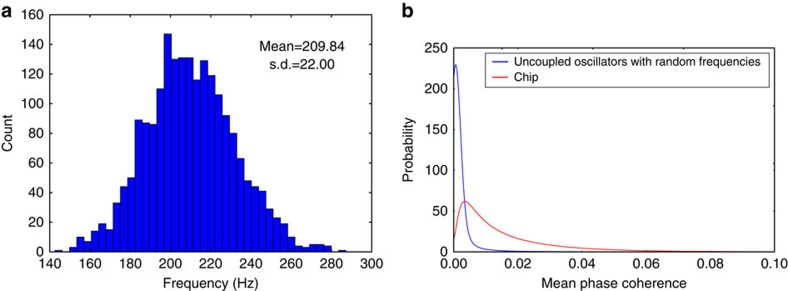
Frequency distribution of chip nodes and internode coherence. (**a**) Frequency distribution of the 2,048 on-chip analogue oscillators for the bias conditions used in the experiments in this paper. (**b**) Normalized distribution of MPC values for all pairs of physical oscillators on the chip (red), and for all pairs of 2,048 artificially generated constant-frequency waveforms (blue).

Figure 8a shows the frequency distribution of the 2,048 on-chip nodes. Due to transistor mismatch, the different neuron circuits have different natural oscillation frequencies for the same current injection. Since the oscillation frequencies are real numbers drawn from a probability distribution arising from the variability inherent in the fabrication process, it is impossible for an oscillator to have a natural frequency that is a rational multiple of another's. Coupling between the oscillators, however, could cause two oscillators with nearby frequencies to lock and effectively have the same frequency. To uncover possible synchronization phenomena among the oscillators, we use the mean phase coherence (MPC) measure, which for two waveforms with instantaneous phases *φ*_1_(*t*) and *φ*_2_(*t*) is defined as:


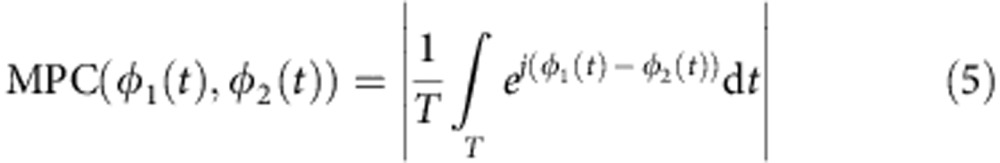


We turned on all 2,048 oscillators and assumed the phase changes linearly from 0 to 2*π* between successive events from an oscillator. We calculated the MPC for each pair of oscillators and the distribution of MPC values is shown in Fig. 8b (red line). In equation (5), we used a time discretization of 0.1 ms and an integration time of 18 s. We randomly generated 2,048 double-precision frequencies from a Gaussian distribution having the same mean and variance as the frequency distribution on the chip and generated an artificial constant-frequency waveform for each of these artificial frequencies. Using the same time discretization and total integration time in equation (5), we evaluated the distribution of MPC values for all possible pairs of the artificial constant-frequency waveforms. Ideally, all these MPC values would be zero, but as shown in Fig. 8b, due to discretization and finite integration time, these uncoupled artificial waveforms have non-zero MPC values. The oscillators on the chip are more synchronized compared with the ideal case (uncoupled oscillators) as evidenced by the heavier tail of their MPC distribution. Oscillator coupling is a potentially serious problem as it compromises the exploration of all possible phase relations, which is key to the exploration of the solution space. Most pairs of on-chip oscillators, however, exhibit very low MPC values that are on par with the MPC values for uncoupled oscillators. Maximum MPC value was 0.34 so no phase locking was observed.

### Mapping 3-SAT problems to the hardware prototype

Each problem variable is represented by one binary node and each 3-SAT constraint/clause is represented by a four-valued node. An event from the 1 port or the 2 port of a binary variable/node denotes that the variable value is 0 or 1, respectively. The constraint/clause node is in state 4 if the constraint is fulfilled, otherwise its state (1 or 2 or 3) denotes which literal in the constraint last emitted an event. Consider a 3-SAT constraint *C*1=(*L*1ν*L*2ν−*L*3). Events from port 2 of variables/nodes *L*1 and *L*2 and events from port 1 of *L*3 should put the *C*1 node at state 4 (constraint fulfilled). A complementary event, that is, an event that does not cause the constraint to be fulfilled (for example, an event from port 2 of *L*3) should do nothing if the constraint is fulfilled as we assume one, or both, of the other two variables fulfil the constraint. However, if the constraint is not fulfilled, a complementary event from the *k*th variable in the constraint should put the constraint node in state *k*.

When the constraint node advertises its state by an event, events from ports 1, 2 or 3 should set the first, second or third variable, respectively, to a constraint-fulfilling state. In the scheme described so far, when a constraint node is fulfilled (in state 4), events from the variables will never move it away from the fulfilled state. To address this, whenever the constraint node generates an event on port 4, this event is routed back to the constraint node and moves it to an arbitrary unfulfilled state (we arbitrarily choose state 3). Thus, within each oscillation cycle of the constraint node, the node has to receive a constraint-fulfilling event from one of its variables to go to state 4 and not to generate an event at the end of the oscillation cycle that forces one of these variables to fulfil the constraint. The constraint nodes were picked from among the nodes with the lowest oscillation frequencies. The globally optimal solution is thus stable as the variable(s) fulfilling a constraint will always be able to generate at least one event that puts the constraint node in a fulfilled state during each cycle of the constraint node.

**Figure 9:**
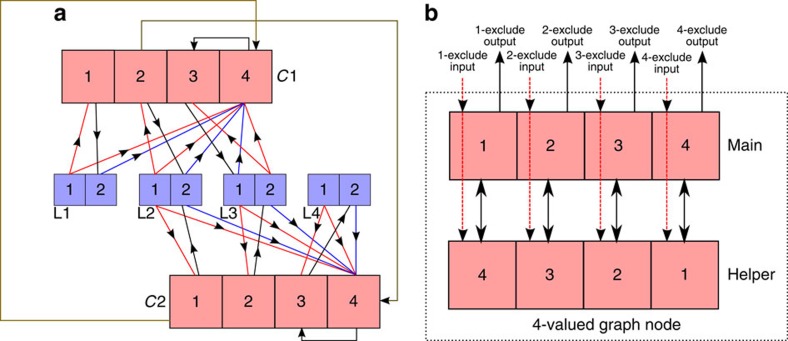
Implementing 3-SAT problems and graph colouring problems on the prototype chip. (**a**) Network implementing the 3-SAT problem *C*1∧*C*2 where *C*1=(*L*1ν*L*2ν−*L*3) and *C*2=(*L*2ν*L*3ν*L*4). Numbered squares indicate the output ports and, indirectly, the input ports of a variable and arrows indicate how events are routed. For example, events from port 1 of *L*1 go to input port 9(‘1001' in binary) of *C*1 that instructs *C*1 to go to state 4 or state 1. Events from port 2 of *L*1 go to port 8 of *C*1 that instructs *C*1 to go to state 4. (**b**) Implementation of a four-colour graph vertex using two four-valued chip nodes that are coupled so that an event from port 1, 2, 3 or 4 of one chip node puts the other node in state 4, 3, 2 or 1, respectively. This vertex receives events from other vertices that go to the exclude input ports of the two chip nodes (red-dashed lines). For example, an event arriving on the one-exclude input port goes to port 14(binary ‘1110') on the ‘main' chip node and port 7(binary ‘0111') on the ‘helper' chip node.

The above scheme is implemented by routing events according to Fig. 9a. Events from variable nodes cannot dislodge a constraint node from the fulfilled state or state 4. Note that state 4 of a constraint node is the lowest priority state according to the state update function in equation (2) so an input event to a constraint node that encodes that state 4 and state *k* (*k*∈{1,2,3}) are allowed will always put the constraint node in state *k*, if it was not already at state 4. If a variable appears with the same sign in multiple constraints (negated or non-negated in all of them), an event generated by one of these constraint nodes that forces this common variable to go to a fulfilling state will automatically fulfil the other constraints as well so we route such events to the other constraints so as to move them to the fulfilled state as shown in Fig. 9a and prevent them from unnecessarily flipping other variables. This scheme for solving 3-SAT is less powerful than the probSAT-based approach described before. At the end of the cycle of an unfulfilled constraint node, the constraint node simply flips the last variable in its domain to generate an event. Due to the continuously shifting phase relations, the choice of which variable to flip is done almost at random with no regard for how many other constraints would be violated due to this flip.

### Mapping graph colouring problems to the hardware prototype

To solve a graph colouring problem on the prototype chip, we can use a simple scheme where a graph vertex is represented by a chip node and events from port *p* of a node (which indicates that the node is in state/colour *p*) go to input port 2^*n*^−1−2^*p*−1^ (all 1s binary string except at position *p*; *p* index starts from 1) of all adjacent nodes/vertices in the graph. We call these input ports the p-exclude input ports as receiving an event on them instructs the node to go to any state except *p*, thereby enforcing the constraint. However, this scheme will not work since a node always goes to an allowed state that has the lowest index when responding to an exclude event (equation (3)). All the nodes would thus quickly get stuck in the 1 and 2 states as the one-exclude and two-exclude events that the nodes send to each other will not be able to move any node out of these 2 states. We use the more elaborate scheme shown in Fig. 9b where two four-valued chip nodes are used to implement one four-valued graph vertex. The value of this graph vertex is index of the last event emitted by the ‘main' chip node.

Pairwise inequality constraints are implemented by routing events from the i-exclude output port of one vertex to the i-exclude input port of the other vertex. Assume a vertex has value 1, that is, the state of the main (helper) chip nodes are 1 (4). The state/colour of this vertex will only change if it receives an event on the one-exclude port. In that case, the ‘main' and ‘helper' chip nodes go to states 2 and 1, respectively, since these are the lowest index allowed states in the two chip nodes. The two chip nodes now have inconsistent states and whichever of them generates an event first forces the other node to switch its state; for example, if the ‘helper' node generates an event first, it forces the ‘main' node to take state 4. A one-exclude input event effectively has a 50% chance of moving this graph node to state 2 and a 50% chance to move it to state 4 due to the irregular phase relations.

The scheme can be extended to six- and eight-valued vertices using three six-valued and four eight-valued chip nodes, respectively, to represent a single graph vertex and it is straightforward to show that using this scheme, the network representing the colouring graph always uses all available colours. Three-, five- and seven-colouring problems can be implemented by adjusting the even colour schemes so that events are routed to input ports that exclude both the colour/index of the source output port, as well as the highest index/colour that will then be unused.

## Additional information

**How to cite this article:** Mostafa, H. *et al.* An event-based architecture for solving constraint satisfaction problems. *Nat. Commun.* 6:8941 doi: 10.1038/ncomms9941 (2015).

## Supplementary Material

Supplementary Information Supplementary Figure 1, Supplementary Note 1 and Supplementary References

## Figures and Tables

**Table 1:** Performance of the graph colouring network.

**Graph**	**No. of vertices**	**No. of edges**	**Density**	**K**	**Number of iterations (GSI)**	**Average number of cycles (network)**
Myciel7	191	2,360	0.13	8	302	**145**
Myciel6	95	755	0.17	7	92	**31**
Myciel5	47	236	0.21	6	97	**19**
Myciel4	23	71	0.28	5	25	**3**
Myciel3	11	20	0.36	4	21	**2**
David	87	986	0.21	11	208	**95**
Anna	138	812	0.21	11	300	**8**
Huck	74	662	0.22	11	84	**8**
Jean	80	508	0.16	10	165	**16**
Queen 5 × 5	25	160	0.53	5	**302**	NA
1_fullins_3	30	100	0.23	4	37	**11**
1_fullins_4	93	593	0.14	5	**76**	366
1_fullins_5	282	3,247	0.08	6	**222**	1,593
2_fullins_3	52	201	0.15	5	67	**47**
2_fullins_4	212	1,621	0.07	6	176	**120**
Miles_250	128	387	0.04	8	**317**	2,021

GSI, gravitational swarm intelligence; NA, not available.

Number of cycles to convergence on common *k*-colouring benchmarks^20^ of our network and a massively parallel algorithm^20^. Each number in the network column is an average of four runs with redrawn oscillator frequencies; one run for the queens graph did not converge in 10^5^ cycles (the other runs averaged 530 steps to convergence). Bold entries indicate better performance.
