# Supplementary material for: An event-based architecture for solving constraint satisfaction problems
Source: Nat Commun. 2015 Dec 8;6:8941. doi: 10.1038/ncomms9941 (PMC4686837; doi:10.1038/ncomms9941)
Supplement: Supplementary Information — Supplementary Figure 1, Supplementary Note 1 and Supplementary References [file ncomms9941-s1.pdf]

Supplementary Figure 1

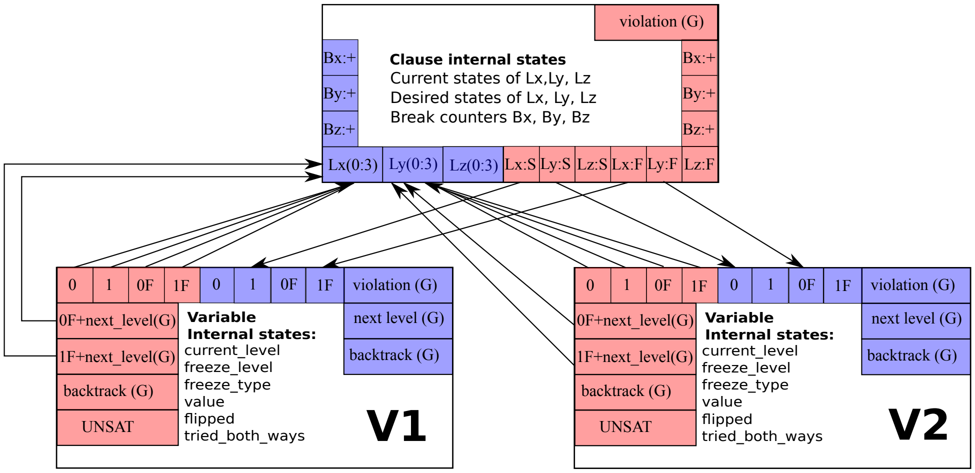

Sample network implementing hybrid probSAT/DPLL The variable and clause nodes implementing the hybrid DPLL/probSAT scheme for the clause $V1 \vee \neg V2$. The squares at the edge of the boxes indicate input ports (blue) and output ports (red). Events are routed along the arrows. Events from output ports with the (G) postfix are routed to all input ports with the same name and the (G) postfix (arrows not shown) of all variables.

Supplementary Note 1. Mapping a complete SAT solver to a network of nodes

Complete algorithms for solving Boolean satisfiability problems typically make use of the DPLL procedure shown in Algorithm 1. This is a recursive sequential procedure where at each recursion level, a decision variable is chosen and assigned a value. The algorithm backtracks to an earlier recursion level if a violation is detected. At the beginning of each recursion level, Boolean constraint propagation (BCP) first identifies all variables whose values are implied by the current partial assignment and adds these variables to the current partial assignment. For example, the only unassigned variable in an unsatisfied clause is assigned a value by BCP to satisfy the clause (unit clause rule). The DPLL algorithm forms the core of many modern SAT solvers. The algorithm is made more efficient through the use of advanced heuristics to pick the literal at each recursion level [1], and most crucially through the use of conflict driven clause learning that augments the original clauses with new clauses when a conflict is detected [2]. This enables later conflicts to be detected earlier in the search tree and thus leads to more efficient pruning of the tree.

We now describe the implementation of a complete SAT solver, that is based on both DPLL and probSAT, on the proposed architecture. The clause and variable nodes are shown in Supplementary Fig.1. Each SAT variable can have four values: 0, 1, 0F, or 1F. The latter two values indicate that the variable value is frozen at 0 or 1 respectively, i.e, it has been fixed either through implication from other frozen variables through BCP, or by being chosen as the decision variable at some DPLL recursion level. The Boolean internal state freeze_type distinguishes between these two cases. Each variable advertises its state each cycle by generating an event on one of the output ports: 0, 1, 0F, or 1F. The current_level internal state is an integer denoting the current DPLL recursion level while freeze_level indicates for frozen variables the recursion level at which they have been frozen.

Algorithm 1 DPLL(F,P)

Input: Formula F, partial assignment P
Output: Satisfying assignment or False
$P_{bcp} \leftarrow P \cup$ **boolean_constraint_propagation**(F, P)
if P_{bcp} is a complete consistent assignment **then**
    return P_{bcp}
else if contradiction(F, P_{bcp}) **then**
    return False
else
    $l \leftarrow$ **select_literal**(F, P_{bcp})
    // short-circuit 'or' operator. Returns non-False argument without casting to Boolean
    return DPLL $(F, P_{bcp} \cup \{l = 1\})$ or **DPLL** $(F, P_{bcp} \cup \{l = 0\})$
end if

Initially, all variables are either 0 or 1, current_level and freeze_level are both zero, and flipped and tried_both_ways are both false. probSAT proceeds as described before. When a variable's advertised state at the end of one cycle is different from its advertised state in the previous cycle (because it has been flipped by a clause), it sets its internal variable flipped to true. At the end of the next cycle and if flipped is still true, the variable freezes (takes the value 0F or 1F), sets its freeze_level to current_level+1 and freeze_type is set to denote that the variable has been frozen because it is the decision variable for a recursion level. The variable generates an event on either "0f+next_level" or " 1f+next_level". This event advertises its frozen state and is routed to the "next_level" input port on all variables which causes all variables to increment their current_level internal counter and reset their flipped internal state to false. tried_both_ways is set to false in the frozen variable. In subsequent cycles, the frozen variable advertises its state through the "0F" or "1F" output ports. Unfrozen variables continue to be flipped through the probSAT dynamics and gradually more of them become frozen and the recursion level (which is stored in the internal counter current_level that is equal in all variables) increases.

Each clause has up-to-date information about the state of the variables in its domain. Unsatisfied clauses flip unfrozen variables in their domains according to the probSAT algorithm. When an unsatisfied clause detects that only one variable in its domain is unfrozen, it sends an event to freeze this variable at a satisfying assignment. This frozen variable sets its freeze_level to the current recursion level (which is available in its current_level counter) and sets its freeze_type to denote that it has been frozen due to the unit clause rule. At some point, an unsatisfied clause node may detect that all its variables are frozen, i.e, a contradiction has been reached. It then generates an event on its "violation" output port which is routed to all variables. All variables frozen through unit clause propagation whose freeze_level is equal to current_level become unfrozen, i.e, their values become either 0 or 1. There is bound to be only one variable frozen as a decision variable whose freeze_level is equal to current_level. If tried_both_ways in this variable is false, this variable simply flips its frozen value and sets tried_both_ways to true. Otherwise, both values have been tried and the variable takes an unfrozen value and generates an event on the "backtrack" output port. All variables receive the backtrack event, and in response decrement the current_level counter. The backtrack event is then treated as a violation event which will cause variables frozen through unit clause propagation at the current level to unfreeze, and a decision variable to either flip or generate further backtrack events. If a frozen decision variable whose freeze_level is 1 (the first decision variable) and which has been tried both ways receives a "violation" or a "backtrack" event when current_level is 1, then it generates an event on the UNSAT port to signal that the problem is unsatisfiable.

This scheme for freezing and unfreezing variables is analogous to the DPLL procedure in Algorithm 1 with one important difference: BCP or unit clause propagation is always active and proceeds in parallel with the mechanism for the selection and freezing of new decision variables. This may cause a violation

caused by the freezing of a decision variable to be detected at deeper/later recursion levels but the violated clause will keep generating violation events until the network has backtracked to the problematic decision variable assignment. A significant part of the execution time of many complete SAT solvers is spent in the unit clause propagation phase. Unit propagation in the described scheme is done in a parallel fashion across all clauses and is thus quite fast. In parallel to the DPLL mechanism, the probSAT mechanism is searching for satisfying assignments in the unfrozen part of the network.

Supplementary References

1. M.W. Moskewicz, C.F. Madigan, Ying Zhao, Lintao Zhang, and Sharad Malik. Chaff: Engineering an efficient sat solver. In *Proceedings of the 38th annual Design Automation Conference*, pages 530–535. ACM, 2001.

2. J.P.M. Silva and K.A. Sakallah. Grasp a new search algorithm for satisfiability. In *Proceedings of the 1996 IEEE/ACM international conference on Computer-aided design*, pages 220–227. IEEE Computer Society, 1997.
